# Effect of Sulforaphane on cardiac injury induced by sepsis in a mouse model: Role of toll-like receptor 4

**DOI:** 10.25122/jml-2023-0015

**Published:** 2023-07

**Authors:** Sarah Mohammed Hussain Hadi, Sahar Majeed, Fadhaa Abdulameer Ghafil, Kaswer Altoraihi, Najah Rayish Hadi

**Affiliations:** 1 Department of Pharmacology and Therapeutics, Faculty of Medicine, University of Kufa, Najaf, Iraq; 2 Medical College, Department of Pharmacology and Therapeutics, University of Kufa, Najaf, Iraq

**Keywords:** sepsis, CLP, cytokines, Oxidative stress, TLR- 4/NF- kB signaling pathways, Sulforaphane, ANOVA: Analysis of Variance, AP-1: Activator protein 1, Bcl-2: B-cell lymphoma 2, °C: Celsius Degree, CLP: Cecal Ligation And Puncture, cTn-I: cardiac troponin I, CK-MB: creatinine kinase MB, DMSO-Dimethyl Sulfoxide, ELISA: enzyme-linked immunosorbent assay, ERK: Extracellular signal-regulated kinase, JNK: c-Jun N-terminal kinase, IL-6: Interleukin 6, IL-10: Interleukin 10, IP: intraperitoneally, LPS: lipopolysaccharide, KEAP-1: Kelch-like ECH-associated protein 1, MAPK: Mitogen-Activated Protein Kinase, MD-2: Myeloid differentiation factor-2, MIF: Macrophage migration inhibitory, NF-κB: Nuclear factor kappa-light-chain-enhancer of activated B cells, Nrf2: nuclear factor erythroid 2-related factor 2, ROS: reactive oxygen species, TNF- α: Tumor necrosis factor alpha, TLR4: Toll-like receptor 4

## Abstract

As sepsis is associated with a 50% increase in mortality, sepsis-induced cardiomyopathy has become a critical topic. A multidisciplinary approach is required for the diagnosis and treatment of septic cardiomyopathy. This study looked at Sulforaphane, a natural product that aims to evaluate cardiac function after sepsis, and its likely mechanism of action. Twenty-four adult male Swiss albino mice were randomly divided into 4 equal groups (n=6): sham, CLP, vehicle Sulforaphane (the same amount of DMSO injected IP one hour before the CLP), and Sulforaphane group (one hour before the CLP, a 5mg/kg dose of Sulforaphane was injected). Cardiac tissue levels of toll-like receptor 4 (TLR-4), pro-inflammatory mediators, anti-inflammatory markers, oxidative stress markers, apoptosis markers, and serum cardiac damage biomarkers were assessed using ELISA. Statistical analyses, including t-tests and ANOVA tests, were performed with a significance level of 0.05 for normally distributed data. Compared to the sham group, the sepsis group had significantly elevated levels of TLR-4, IL-6, TNF-α, MIF, F2-isoprostane, caspase-3, cTn-I, and CK-MB (p<0.05). In contrast, the Sulforaphane pre-treated group demonstrated significantly lower levels of these markers (p<0.05). Additionally, Bcl-2 levels were significantly reduced (p<0.05) in the Sulforaphane group. Sulforaphane administration also significantly attenuated cardiac tissue injury (p<0.05). The findings suggest that Sulforaphane can decrease heart damage in male mice during CLP-induced polymicrobial sepsis by suppressing TLR-4/NF-kB downstream signal transduction pathways.

## INTRODUCTION

Recent definitions of “sepsis” include systemic inflammatory response syndrome (SIRS), which develops as a result of infection and results in organ dysfunction. It is frequently accompanied by a cytokine storm in which neutrophils and macrophages release a large number of cytokines, including TNF-α, prostaglandins, and interleukins [1, 2]. Numerous studies have demonstrated that the mortality rate rises when sepsis develops into septic shock, a life-threatening condition marked by persistent hypotension that does not respond to resuscitation fluids and inotropic or vasopressor medications [3]. On the other hand, septic cardiomyopathy (SCM), an acute illness resulting from sepsis, can lead to cardiac failure. Early detection and diagnosis of SCM are crucial to initiate appropriate interventions and restore heart function [4]. Numerous studies reported incidences ranging from 13.8 to 40%, and the death rate increases by two to three times, reaching 70 to 90%, in people with SCM. However, the lack of comprehensive investigations and standardized diagnostic criteria has resulted in challenges in accurately estimating the prevalence of septic cardiomyopathy [5]. Toll-like receptors are transmembrane proteins having an extracellular amino-terminal involved in ligand binding, and a cytoplasmic carboxyl-terminal is known as toll interleukin1 receptor (TIR) for signal transduction [6]. These receptors get activated after they establish a connection with their ligands, either internal damage-associated molecular patterns (DAMPS) or external pathogen-related molecular patterns (PAMPS). This binding causes the creation of proinflammatory cytokines and interferon I, which starts a series of signal transduction pathways [7]. The innate immune system relies heavily on toll-like receptors (TLRs) to activate signaling cascades, such as the nuclear factor kappa B (NF-κB) and inflammasome pathways [8], upon recognition of specific ligands from invading pathogens or damaged cells. This recognition leads to the production of proinflammatory cytokines and chemokines [1]. As mentioned above, sepsis has an abnormally high level of cytokine release. Immune cells produce cytokines, which are small soluble glycoproteins. Tumor necrosis factor (TNF-α), interleukin-1 (IL-1), interleukin-6 (IL-6), interleukin-8 (IL-8), interferon (IFN), and macrophage migration inhibitory factor (MIF) are proinflammatory mediators, while interleukin-10 (IL-10) and IL-4 are anti-inflammatory mediators. Together, these two cytokines regulate the inflammatory response [9]. Cellular changes, including nuclear degeneration, chromatin condensation, DNA oxidation, and cell residue phagocytosis, are all part of apoptosis, also known as programmed cell death [10]. These metabolic processes might be carried out via intrinsic or extrinsic mechanisms [11]. It has been demonstrated that the antioxidant Sulforaphane, obtained from cruciferous vegetables, has protective benefits on many organs, including the liver, lungs, and hearts. Sulforaphane effects on sepsis-induced SCM were the focus of this investigation [12].

## MATERIAL AND METHODS

The research was conducted at the Faculty of Medicine/University of Kufa, Department of Pharmacology and Therapeutics, and the Middle Euphrates Unit for Cancer Researches.

### Study design

To minimize physiological variability associated with the estrous cycle of female mice, 24 adult male Swiss albino mice, aged 6-8 weeks and weighing 20-30g, were obtained from the animal house at the College of Science, Kufa University. The mice were housed in cages with a temperature of 25°C, a humidity level of around 60-65%, and 12h light/12h dark cycles at the animal home, where they had unlimited access to food and drink. The mice were divided into four groups (n=6 per group):

1. Sham group (negative control): Mice in this group underwent laparotomy without the cecal ligation and puncture (CLP) procedure.
2. Cecal ligation and puncture group (CLP) (positive control): Mice in this group underwent the CLP procedure to induce sepsis [13].
3. Vehicle Sulforaphane group: Mice in this group received the same amount of dimethyl sulfoxide (DMSO) injected intraperitoneally one hour before the CLP procedure [10].
4. Sulforaphane pretreated group: Mice in this group received a 5 mg/kg dose of Sulforaphane injected intraperitoneally one hour before the CLP procedure, based on a previous study [10].

### Experimental procedure

The mice were anesthetized with a combination of xylazine (10 mg/kg) and ketamine (100 mg/kg) administered intraperitoneally (IP) [14]. The CLP (cecal ligation and puncture) sepsis model was chosen for this study due to its high stability, reproducibility, and relevance to the nature and progression of severe sepsis in humans [15]. The CLP procedure involves performing an abdominal laparotomy with a 1.5 cm midline incision to expose the cecum. The cecum was then ligated, followed by two punctures with a G-21 needle just below the ileocecal valve. Afterward, a small amount of fecal material was cautiously squeezed through the puncture sites on both sides. After ligating and puncturing the cecum, it was placed back into the abdominal cavity, and the incision was sutured with a 3.0 surgical suture. Subcutaneous administration of 1 ml saline solution was performed, and the mice were allowed to recover in their cages [12].

### Preparation of Sulforaphane

Sulforaphane was purchased from ChemScene and prepared in diluted DMSO before being administered intraperitoneally at a dosage of 5 mg/kg 1 hour before the CLP procedure [10].

### Preparation of samples

#### Blood samples

Blood samples were collected from the mice while they were under terminal anesthesia. An appropriate needle was used to puncture the mice's hearts and obtain a considerable volume of high-quality blood. The blood was collected into gel tubes to obtain serum for the required tests. After allowing the samples to settle for 10-20 minutes at ambient temperature, they were centrifuged at 2000 RPM for 20 minutes at 4°C. The supernatant was carefully collected without any sediment. The collected serum samples were stored at -80°C for further testing. ELISA analysis was performed on the blood samples to examine the levels of cardiac troponin I (cTn-I) and creatinine kinase MB (CK-MB) [16].

#### Tissue samples

Tissue samples from the hearts were divided into two halves. One half was frozen for ELISA analysis, and the other half was immediately preserved for histopathological examination.

### Histopathological examination

Tissue samples were fixed in 10% neutral formalin. Paraffin-embedded samples were sectioned into 4-5 µm thick slices using a microtome. The sections were stained with hematoxylin and eosin (H&E) [17]. The degree of heart damage was assessed, and photographs were taken using an optical microscope (n=5 sections per heart). Following Zingarelli's protocol, histological sections from all groups were assessed and graded to roughly estimate the variance in heart damage [18]. The scoring system included four levels of severity: 0 (normal tissue), 1 (mild interstitial edema and localized necrosis), 2 (moderate myocardial cell swelling and diffuse necrosis), 3 (severe ischemia and neutrophil buildup), and 4 (very severe with contraction bands, leukocyte infiltration, ischemia, and hemorrhage) [19, 20].

### ELISA analysis

Myocardial tissues were washed in ice-cold water to remove remaining red cells or blood clots. The tissues were homogenized in phosphate-buffered saline (PBS) containing 0.5% Triton X-100 and a protease inhibitor cocktail. The homogenates were centrifuged at 4°C for 20 minutes at 3000 rpm, and the supernatant was utilized for ELISA analysis. Commercial ELISA kits (Elabscience Technology Laboratory) were employed to measure the levels of macrophage migration inhibitory factor (MIF), tumor necrosis factor-α (TNF-α), interleukin-10 (IL-10), interleukin-6 (IL-6), caspase-3, F2-isoprostane, Bcl-2, and toll-like receptor 4 (TLR-4) in myocardial tissue.

### Statistical analysis

Statistical analysis was performed using SPSS version 26. The normal distribution of data was assessed using the Kolmogorov-Smirnov and Shapiro tests. For normally distributed data, t-tests and ANOVA tests were conducted with a significance level of 0.05.

## RESULTS

**Figure 1:**
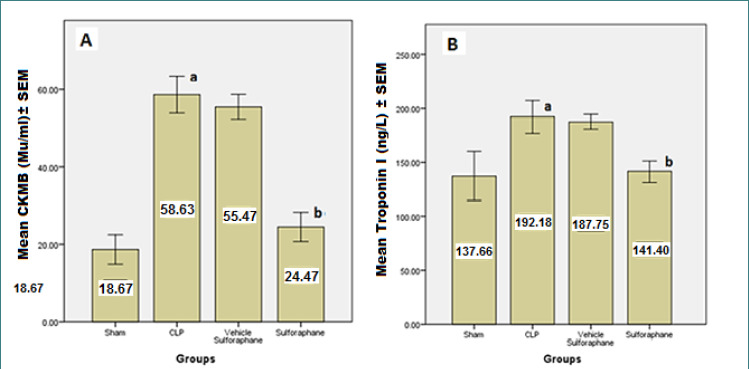
Comparison of mean (A) CKMB±SEM and (B) cardiac troponin I (cTn-I) ±SEM among the four experimental groups at the end of the experiment; (a) significant difference in the CLP group compared to the sham group (p<0.05), (b) significant difference in the Sulforaphane group compared to the CLP group (p<0.05)

**Figure 2:**
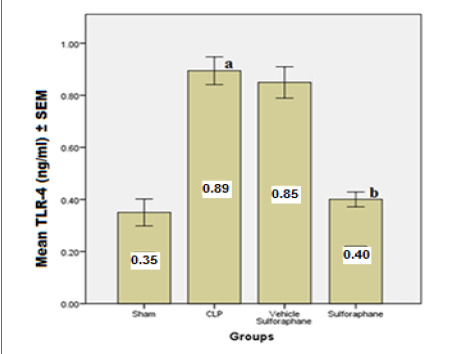
Comparison of mean TLR-4 levels ±SEM among the four experimental groups at the end of the experiment; (a) significant difference in the CLP group compared to the sham group (p<0.05), (b) significant difference in the Sulforaphane group compared to the CLP group (p<0.05)

**Figure 3:**
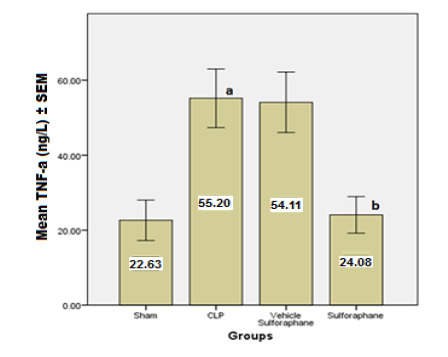
Comparison of mean TNF-a levels ±SEM among the four experimental groups at the end of the experiment; (a) significant difference in the CLP group compared to the sham group (p<0.05), (b) significant difference in the Sulforaphane group compared to the CLP group (p<0.05)

**Figure 4:**
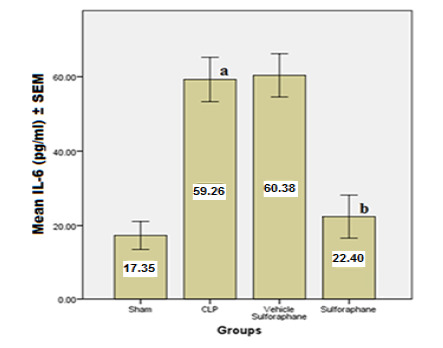
Comparison of mean IL-6 levels ±SEM among the four experimental groups at the end of the experiment; (a) significant difference in the CLP group compared to the sham group (p<0.05), (b) significant difference in the Sulforaphane group compared to the CLP group (p<0.05)

**Figure 5:**
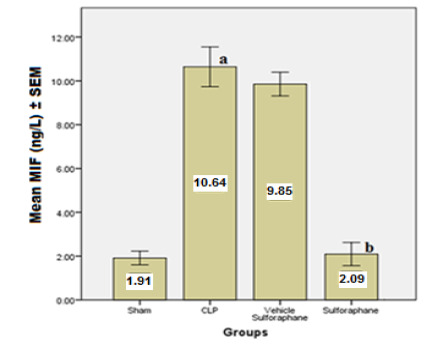
Comparison of mean MIF levels ±SEM among the four experimental groups at the end of the experiment; (a) significant difference in the CLP group compared to the sham group (p<0.05), (b) significant difference in the Sulforaphane group compared to the CLP group (p<0.05)

**Figure 6:**
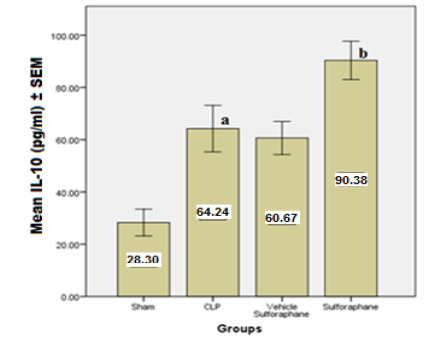
Comparison of mean IL-10 levels ±SEM among the four experimental groups at the end of the experiment; (a) significant difference in CLP group compared to the sham group (p<0.05), (b) significant difference in the Sulforaphane group compared to the CLP group (p<0.05)

**Figure 7:**
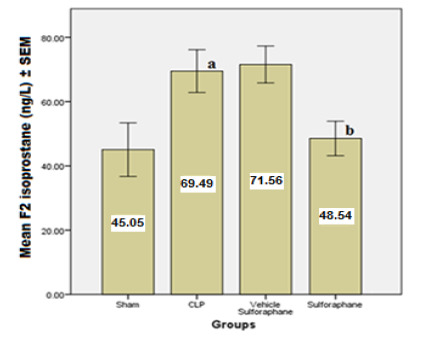
Comparison of mean F2-isoprostane levels ± SEM among the four experimental groups at the end of the experiment; (a) significant difference in CLP group compared to the sham group (p<0.05), (b) significant difference in the Sulforaphane group compared to the CLP group (p<0.05)

**Figure 8:**
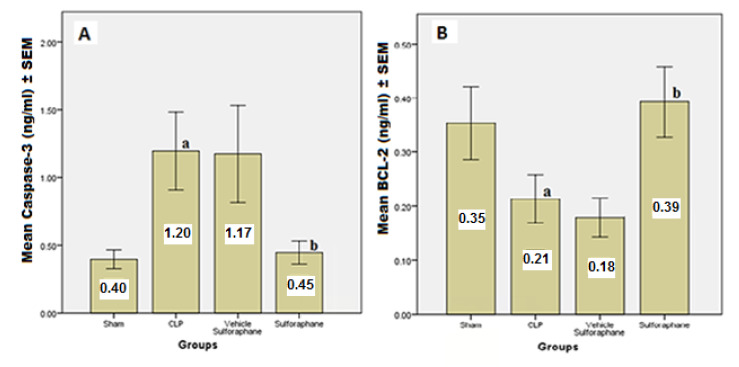
Comparison of mean (A) caspase-3 and (B) Bcl-2 levels ± SEM among the four experimental groups at the end of the experiment; (a) significant difference in CLP group compared to the sham group (p<0.05), (b) significant difference in the Sulforaphane group compared to the CLP group (p<0.05)

According to the data, serum concentrations of cardiac troponin I (cTn-I) and creatinine kinase MB (CK-MB) (Figure 1 A-B), two biomarkers that indicate myocardial injury, were significantly reduced by Sulforaphane. Additionally, the expression of toll-like receptor 4 (TLR4) was significantly reduced (Figure 2). Furthermore, Sulforaphane treatment considerably reduced inflammation in the cardiac tissue by lowering pro-inflammatory TNF-a (Figure 3), IL-6 (Figure 4), and MIF (Figure 5) markers while increasing anti-inflammatory IL-10 (Figure 6) indicators. Sulforaphane also decreased the oxidative stress marker F2-isoprostane (Figure 7) and the pro-apoptotic marker caspase-3 while increasing the anti-apoptotic marker Bcl-2 (Figure 8 A-B).

### Histological findings

**Figure 9:**
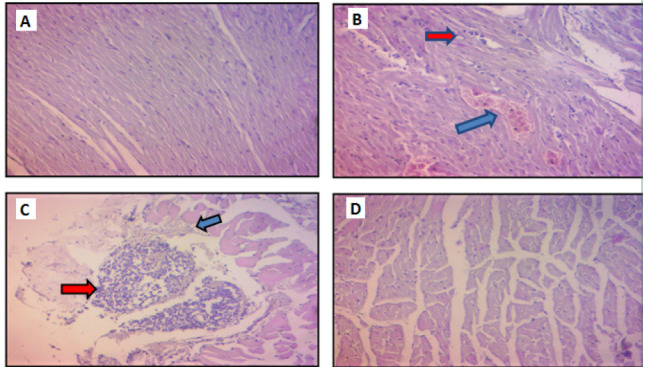
Histopathological examination of myocardial tissue. A) Myocardial tissue from the sham group demonstrating normal-looking cardiac tissue. B) Myocardial tissue from the CLP group showing severe myocardial damage (score 4). C) Myocardial tissue from the Vehicle Sulforaphane group exhibiting significant myocardial damage (score 4). D) Myocardial tissue from the Sulforaphane group displaying mild myocardial damage (score 1). Histological sections were stained with H&E (40X). Blue arrows indicate areas with hemorrhage, while red arrows indicate areas with acute inflammatory cells

Histological examination of the myocardial tissue revealed varying levels of damage among the experimental groups. The sham group exhibited normal myocardial tissue. In contrast, the CLP group, which experienced sepsis induction, showed significant myocardial damage with a score of 4. This damage was characterized by the presence of contraction bands, infiltration of polymorphonuclear leukocytes (PMN), interstitial edema, and localized extravasation of red blood cells. Similarly, the vehicle Sulforaphane group exhibited significant myocardial damage with a score of 4. In contrast, the Sulforaphane group demonstrated milder myocardial damage with a score of 1, indicating only slight differences compared to the sham group. Histological findings are shown in Figure 9 A-D.

## DISCUSSION

Sepsis is defined as a series of events that can progress from multiorgan dysfunction to failure and death [21]. The heart is commonly affected by sepsis, and myocardial dysfunction is observed in approximately 50% of septic patients [22]. TLR-4 has been extensively studied in the context of cardiac function. In mice, the absence of a functional TLR-4 gene has been associated with protection against reduced myocardial shortening, indicating improved cardiac contractility [23]. TLR-4 plays a crucial role in activating nuclear factor kappa B (NF-κB), a transcription factor that regulates the expression of inflammatory cytokine genes. TLR-4-mediated signaling leads to the activation of NF-κB, triggering inflammation, reactive oxygen species (ROS) generation, programmed cell death, and intracellular calcium accumulation. After a series of sequential events, NF-κB is activated, which causes the release of inflammatory cytokines like TNF-α and IL-6 that restrict Ca2+ migration, control the reactive oxygen species pathway, and degrade essential proteins [24].

In sepsis, the heart can experience myocardial damage, leading to increased biomarkers associated with cardiac injury, such as creatinine kinase MB (CK-MB) and cardiac troponin I (cTn-I). However, in our study, the group pre-treated with Sulforaphane exhibited a significant decrease in these biomarkers. This reduction in biomarker levels can be attributed to the antagonistic action of Sulforaphane on TLR4 and the inhibition of NF-κB pathway [25]. TLR-4-dependent pathways can be activated by lipopolysaccharide (LPS) and endogenous substances generated during sepsis, as seen in the CLP, which increased the expression of TLR4 [26]. In contrast, Sulforaphane pre-treatment reduced TLR-4 expression by interacting with cysteine residues in the extracellular domain of TLR-4, leading to the generation of adducts that decrease receptor oligomerization. This interaction is thiol-dependent and reduces NF-κB activation in macrophages. In addition, Sulforaphane competes with LPS for binding to MD-2, a hydrophobic pocket that aids TLR-4 dimerization, thereby inhibiting LPS binding to the TLR4/MD-2 complex [27, 28]. The inhibition of TLR-4 by Sulforaphane leads to a decrease in activator protein 1 (AP-1) and NF-κB, resulting in the downregulation of pro-inflammatory cytokines such as TNF-α, IL-6, and macrophage migration inhibitory factor (MIF) [29, 30].

Furthermore, Sulforaphane activates nuclear factor erythroid 2-related factor 2 (Nrf2), which regulates antioxidant and anti-inflammatory responses. Our results suggest that the overexpression of anti-inflammatory cytokine IL-10 and downregulation of pro-inflammatory cytokines are mediated by Sulforaphane-induced Nrf2 activation, which is regulated by the inhibition of the c-Jun N-terminal kinase (JNK) pathway [26]. Numerous studies have demonstrated that in sepsis, antioxidant enzyme activity declines while ROS/RNS production increases; this imbalance is known as oxidative stress [31, 32]. The oxidation-reduction balance is maintained by various processes, including the Keap1-Nrf2 and NF-κB pathways. Measurements of lipid peroxidation products (F2-isoprostane) generated by free radicals are often used [33]. The current study demonstrates a relationship between oxidative stress reduction and Sulforaphane administration, possibly due to targeting the Nrf2 pathway. In addition to the direct anti-oxidant action mediated via NRF2 signaling, Sulforaphane may impede the redox-sensitive DNA binding and the upregulation of the NF-κB, the pro-inflammatory transcription factor [34]. During sepsis, LPS binding to TLR-4 may activate the JNK, ERK, or p38 MAPK pathways or the mitochondria-mediated intrinsic apoptosis pathway, which in turn activates caspase-3, an apoptosis activator effector, and downregulates Bcl-2, an inhibitor of apoptosis [35]. In our study, we observed a decrease in caspase-3 levels and an increase in Bcl-2 levels in the Sulforaphane pre-treated group, indicating inhibition of apoptosis. This effect may be attributed to the suppression of the MAPK inflammatory pathway, which leads to decreased transcription of pro-inflammatory cytokine genes, as well as potential interference with LPS detection by TLR-4 and suppression of the NF-κB pathway [36, 37].

## CONCLUSION

Our findings support the notion that Sulforaphane has significant anti-inflammatory, anti-oxidative, and anti-apoptotic effects because of its strong inhibitory effect on TLR4/NF-κB signaling pathways, which reduces the heart damage caused by sepsis.
